# Editorial: Emerging Approaches for Typing, Detection, Characterization, and Traceback of *Escherichia coli*

**DOI:** 10.3389/fmicb.2016.02089

**Published:** 2016-12-27

**Authors:** Pina M. Fratamico, Chitrita DebRoy, David S. Needleman

**Affiliations:** ^1^Agricultural Research Service, United States Department of Agriculture, Eastern Regional Research CenterWyndmoor, PA, USA; ^2^Department of Veterinary and Biomedical Sciences, E. coli Reference Center, The Pennsylvania State University, University ParkPA, USA

**Keywords:** *Escherichia coli*, virulence factors, whole genome sequencing, serotyping, detection, subtyping

Commensal *E. coli* inhabit the large intestines of humans and animals and are important in maintaining normal intestinal homeostasis. There are also many groups of disease-causing *E. coli*, including diarrheagenic and extra-intestinal pathogenic *E. coli* (ExPEC). *E. coli* strains have been identified primarily based on their O- and H-antigens, defining the *E. coli* serotype. There are approximately 188 somatic O-antigens, 74 capsular K-antigens, 53 flagellar H-antigens, and greater than 60 fimbrial F-antigens in *E. coli* identified based on antigens that produce an immune response in animals. This research topic consists of articles based on the subject of an international workshop on “Emerging approaches for typing, detection and characterization of *Escherichia coli”* held at the Pennsylvania State University in 2015. This workshop brought together well-known scientists from throughout the world to provide a forum for the exchange of ideas with regard to examining the current serotype classification and nomenclature for *E. coli*, emerging pathotypes, and new technologies and whole genome sequencing (WGS) for detection, characterization, and outbreak investigation. Scientists who presented papers at the conference were affiliated with public health laboratories, regulatory agencies, academic institutions, and industry groups from the U.S., United Kingdom, France, Italy, Canada, Denmark, Germany, and Japan, as well as industry groups working on technologies for characterization, detection, identification, and subtyping *E. coli*. This workshop provided a forum to discuss different concepts and practices for typing *E. coli* based on O- and H-antigens and for characterization of pathotypes. Furthermore, there were discussions on the progress made in the area of WGS as a tool for *E. coli* typing, subtyping, characterization, diagnostics, and outbreak investigation, as well as on the evolution and emergence of highly pathogenic strains (Franz et al., 2014).

A mini review, providing an overview on this research topic is “Advances in molecular serotyping and subtyping of *Escherichia coli*” (Fratamico et al.). Phenotypic methods, such as serotyping for O- and H-antigen determination, biotyping based on biochemical characteristics, or bacteriophage typing have been used for differentiating and characterizing *E. coli* for many years; however, these methods are labor intensive, time consuming, and not always accurate. Recent advances in molecular techniques and DNA sequencing technologies have led to the development of typing and subtyping methods for *E. coli* that are more accurate and have better discriminatory power than phenotypic typing methods. WGS of *E. coli* is allowing more rapid and accurate identification of pathogenic strains and is replacing pulsed-field gel electrophoresis (PFGE), a gold standard for investigating food-borne disease outbreaks (Franz et al., 2014; Joensen et al., 2014). The necessity to analyze large amounts of data generated from WGS has also led to the development of new and more efficient bioinformatics pipelines. Lindsey et al. described and validated the use of a genotyping plug-in within BioNumerics® v7.5 to provide an accurate and cost-effective single workflow to replace the complex suite of workflows currently used to perform the bioinformatics analyses of WGS data.

Parsons et al. presented a review of various methods and strategies for detection and characterization of Shiga toxin-producing *E. coli* (STEC), including the use of various chromogenic agars, enzyme immunoassays, and qPCR. Their group at the Provincial Laboratory of Public Health in Canada have also utilized WGS, single nucleotide polymorphism (SNP), and k-mer analysis for epidemiological investigations, and they compared these methods to pulsed-field gel electrophoresis and multiple-locus variable number tandem repeat analysis. In agreement with other studies, they found that the sequence-based methods offer higher resolution (Salipante et al., 2015). Use of WGS to distinguish *E. coli* O157:H7 outbreak strains was presented by Rusconi et al. Utilizing customized high-resolution bioinformatics sequence typing approaches, the core genomes, mobilome plasticity, and SNPs were determined. In addition to providing higher strain discriminatory power compared to currently used methods such as PFGE, sequence-based strategies offer the advantages of higher throughput and improved cost-effectiveness. Whole genome sequencing was compared to phenotypic serotyping results on non-O157 STEC isolated from human fecal specimens (Chattaway et al.). They found that most isolates for which the O-group could not be identified by serotyping could be O-typed using WGS data. Furthermore, WGS data provided more accurate *stx*-subtyping results compared to PCR. Thus, WGS provided more reliable results for strain identification and characterization compared to traditional serotyping and PCR, enabling a higher level of strain discrimination and the ability to predict the pathogenic potential.

WGS data on enteric pathogens is shedding light into the relationship between *Shigella* species and *E. coli*. *Shigella* species and enteroinvasive *E. coli* (EIEC) are very similar genetically, and it has been proposed that *Shigella* and EIEC be classified as a single pathovar of *E. coli*. The work of Pettengill et al. based on WGS data and phylogenetic analyses of EIEC and *Shigella* also did not support distinct genera designations for these organisms. They suggested that *Shigella* be classified as EIEC, and common O-antigen designations should be used. They also identified a panel of 404 SNP markers that could be used to discriminate among different phylogenetic clades.

Targeted sequencing in combination with PCR has furthered the characterization and typing of *E. coli*. The sequences of the O-antigen gene clusters of 6 STEC O-groups that were found to be non-typeable using a previously reported *E. coli* O-genotyping PCR system were published by Iguchi et al. They then developed specific PCR assays to identify these novel O-groups that they designated as OgN1, OgN8, OgN9, OgN10, OgN12, and OgN31. Therefore, many O-groups that are not able to be identified by traditional serotyping or other methods may represent new *E. coli* O-groups and will require new O-group designations. For distinguishing the H1 and H12 H-types of *E. coli* that have 97.5% identity in their *fliC*_H1_ and *fliC*_H12_ genes, Beutin et al. analyzed the sequences of *E. coli* H1 and H12 strains and developed a two-step real-time PCR detection procedure where the first step PCR assay would detect both H1 and H12 flagellar types followed by second step real-time PCR that discriminated H1 or H12. These technologies will ultimately lead to further clarification *E. coli* O- and H-genotypes and in their phylogenetic relationships.

In an effort to develop more accurate and specific methods for STEC detection, Delannoy et al. tested beef enrichments for the presence of specific STEC genetic markers and found that *stx, eae, espK*, and *espV*, in combination with CRISPR_O26:H11_ served as a suitable set of markers for screening for STEC of concern in beef enrichments, thus lowering the number of samples that required additional testing. A High Resolution Virulence Allelic Profiling (HReVAP) approach to determine allelic variants in genes carried on STEC pathogenicity islands (LEE locus, OI-122, and OI-57) was developed by Michelacci et al. Following cluster analysis of the allelic forms of 91 virulence genes, representing allelic signatures based on HReVAP analyses, they investigated the phylogeny of STEC and identified subpopulations within groups. The approach provided evidence for co-evolution of LEE and OI-122, likely acquired through a single event, and it could be used a tool for studying the evolution of STEC mobile genetic elements. Knowledge of allelic signatures in STEC isolates and the presence of specific genes associated with strains that cause severe illness (hemorrhagic colitis and hemolytic uremic syndrome) improves the ability to test for and identify STEC strains of greater clinical significance.

Knowledge of the virulence genes of food-borne pathogens carried by animals provides information to assess their genetic diversity and zoonotic potential. Baranzoni et al. characterized STEC strains in pigs and found that strains that carried *stx*2e (81%) were the more predominant STEC, followed by strains that carried *stx*1a (14%), *stx*2d (3%), and *stx*1c (1%) as determined by PCR and an *E. coli* Identification (ECID) Array. The swine isolates also carried other virulence genes such as *iha, lpfA*O26, *lpfA*O157, *fedA, orfA*, and *orfB*. The work presented new insights into the virulence genes associated with porcine isolates that may potentially cause human illness. STEC prevalence throughout the pork production chain in Argentina was studied by Colello et al. by determining the O-groups and virulence gene content of strains collected from farms, during slaughter and processing, and at retail markets. The prevalence and characterization of STEC strains throughout the system reflected vertical transmission of the strains, emphasizing the importance of integrated STEC control systems from farm to table.

Overuse and misuse of antibiotics in humans and animals has led to an increase in antibiotic resistant bacteria, which is a major public health concern worldwide. The prevalence of extended-spectrum beta-lactamase (ESBL)-producing *E. coli* in dairy cattle farms in Egypt was studied by Braun et al. using DNA microarray-based assays that detect resistance genes, and the isolated strains were also serotyped using a SeroGenoTyping array. The study showed a notable prevalence of ESBL-producing strains, and carbapenemase genes (*bla*OXA-48 and *bla*OXA-181) (encode enzymes in the beta-lactamase family) were also detected in isolates resistant to imipenem and meropenem. The high prevalence of antibiotic resistant *E. coli* strains in cattle farms in Egypt points to the importance of increased surveillance efforts in developing countries. Another array-based approach for STEC characterization was reviewed by Carter et al. The “suspension array” technology, which can potentially analyze up to 500 targets in one reaction, was used in both multiplex PCR and immunoassay microbead-based formats to detect *E. coli* serogroup-specific markers, as well as virulence markers. In addition to virulence- and serotype-specific genes, high-throughput array-based systems can potentially detect antibiotic resistance determinants, as well.

Cattle are an important reservoir of STEC, and STEC that cause serious human illness can be found in higher rates from veal calves compared to beef cattle. Methods for determining the prevalence of STEC in veal hides and carcasses, as well as for enumeration of the STEC were evaluated. The methods included a molecular-based most probable number method (MPN), a qPCR assay, and a digital PCR assay (Luedtke and Bosilevac). Each molecular method had strengths and weaknesses related to the detection and enumeration rate and dynamic range for enumeration. ExPEC cause the majority of urinary tract infections and are associated with many other types of extraintestinal infections. Food, particularly poultry, can carry ExPEC, particularly antibiotic-resistant strains that cause human illness (Manges, 2016). Sommers et al. presented information on interventions for control of uropathogenic *E. coli* (UPEC) in ground chicken meat, purge, and chicken meat surfaces. Using non-thermal processing technologies such as high pressure processing, gamma irradiation, and ultraviolet light (UV-C) they showed significant reduction in the levels of UPEC strains in poultry meat; therefore, these treatments may be useful for the production of safer food products, particularly for at-risk consumers.

In summary, many new technologies have become available to enhance the ability to detect, identify, and characterize *E. coli*. WGS in particular is providing a powerful and expanding range of information to identify targets for development of improved intervention strategies and is being implemented for source tracking and as part of routine surveillance systems (Bergholz et al., 2014). We expect that further developments in WGS and other genomic and molecular technologies will continue to contribute to a greater understanding of the pathogenesis of *E. coli* and ultimately provide better resources for improving public health.

## Author contributions

All authors listed, have made substantial, direct and intellectual contribution to the work, and approved it for publication.

### Conflict of interest statement

The authors declare that the research was conducted in the absence of any commercial or financial relationships that could be construed as a potential conflict of interest.
